# Muscle deoxygenation during ramp incremental cycle exercise in older adults with type 2 diabetes

**DOI:** 10.1007/s00421-023-05297-y

**Published:** 2023-08-28

**Authors:** Adam McDermott, Aaron Nevin, Norita Gildea, Joel Rocha, Donal O’Shea, Mikel Egaña

**Affiliations:** 1 Department of Physiology, School of Medicine, Trinity College Dublin, The University of Dublin, Dublin 2, Ireland; 2 Dundee, UK; 3 Endocrinology, St Columcille’s and St Vincent’s Hospitals, Dublin, Ireland

**Keywords:** Near-infrared spectroscopy, Oxygen extraction, Cycling, Exercise tolerance, Type 2 diabetes, Ageing

## Abstract

**Purpose:**

To explore profiles of fractional O_2_ extraction (using near-infrared spectroscopy) during ramp incremental cycling in older individuals with type 2 diabetes (T2D).

**Methods:**

Twelve individuals with T2D (mean ± SD, age: 63 ± 3 years) and 12 healthy controls (mean age: 65 ± 3 years) completed a ramp cycling exercise. Rates of muscle deoxygenation (i.e., deoxygenated haemoglobin and myoglobin, Δ[HHb + Mb]) profiles of the vastus lateralis muscle were normalised to 100% of the response, plotted against absolute (W) and relative (%_peak_) power output (PO) and fitted with a double linear regression model.

**Results:**

Peak oxygen uptake (V̇O_2peak_) was significantly (*P* < 0.01) reduced in T2D (23.0 ± 4.2 ml.kg^−1^.min^−1^) compared with controls (28.3 ± 5.3 ml.kg^−1^.min^−1^). The slope of the first linear segment of the model was greater (median (interquartile range)) in T2D (1.06 (1.50)) than controls (0.79 (1.06)) when Δ%[HHb + Mb] was plotted as a function of PO. In addition, the onset of the second linear segment of the Δ%[HHb + Mb]/PO model occurred at a lower exercise intensity in T2D (101 ± 35 W) than controls (140 ± 34 W) and it displayed a near-plateau response in both groups. When the relationship of the Δ%[HHb + Mb] profile was expressed as a function of %PO no differences were observed in any parameters of the double linear model.

**Conclusions:**

These findings suggest that older individuals with uncomplicated T2D demonstrate greater fractional oxygen extraction for a given absolute PO compared with older controls. Thus, the reductions in V̇O_2peak_ in older people with T2D are likely influenced by impairments in microvascular O_2_ delivery.

## Introduction

Adult individuals with uncomplicated type 2 diabetes (T2D) of all ages demonstrate an impaired peak aerobic exercise capacity, characterised by a significant reduction (ranging from ~ 12 to  30%) in peak oxygen uptake (V̇O_2peak_) (Mac Ananey et al. 2011; O'Connor et al. 2015, 2012; Kiely et al. 2015; Regensteiner et al. 1998). This decrement is clinically significant given that V̇O_2peak_ is an established clinical predictor of cardiovascular and all-cause mortality (Wei et al. 2000). While the mechanisms for the reduced V̇O_2peak_ responses in T2D are inconclusive, important mediators include systemic cardiovascular limitations (e.g., impaired left ventricular filling (Wilson et al. 2017a, 2017b)), reductions in peripheral vasodilation and/or microvascular dysfunction in the lower limbs (Gildea et al. 2021c; Rocha et al. 2022; Gildea et al. 2019; Rocha et al. 2019; Poitras et al. 2015; MacAnaney et al. 2011), or decreased mitochondrial content and function (Ritov et al. 2005; Boushel et al. 2007).

The regulation of maximum V̇O_2_ is influenced by the ability of the body to uptake and efficiently deliver O_2_ to the skeletal muscle mitochondria to carry out oxidative processes (Poole 1997; Wagner et al. 1997), and is therefore, a product of whole body perfusive and diffusive O_2_ conductance as represented by the Fick equation. According to the Fick equation, maximum V̇O_2_ is governed by changes in cardiac output and arteriovenous oxygen difference (a-vO_2diff_), thus, providing a volume-weighted average of fractional O_2_ extraction across the whole body when measured at the pulmonary level. A limitation of this approach is that during exercise, changes in pulmonary V̇O_2_ may not accurately reflect changes in microvascular V̇O_2_ within the working skeletal muscle given the constant adjustments of muscle microvascular blood flow and O_2_ extraction owing to the redistribution of blood flow (Spencer et al. 2012; Iannetta et al. 2017; Okushima et al. 2016). In this regard, while peak a-vO_2diff_ in T2D does not seem to be altered during cycling ramp exercise (Green et al. 2015), Gildea et al. (Gildea et al. 2019) recently reported a greater reliance on fractional oxygen extraction during ramp incremental cycling exercise in middle-aged individuals with T2D (mean age 48 yr, range 36–55 yr) compared with healthy controls which was accompanied by a significant reduction in V̇O_2peak_. This was represented by an increased near-infrared spectroscopy (NIRS)-derived rate of muscle deoxygenation (i.e., deoxygenated haemoglobin and myoglobin, [Δ%HHb + Mb]) of the vastus lateralis (VL) plotted against % power output (PO). This suggests that impaired microvascular blood flow responses contributed, at least partly, to the exercise intolerance observed in middle-aged individuals with T2D (Gildea et al. 2019). Consistent with this notion, a greater rate of muscle deoxygenation during ramp exercise has also been reported when exercising in the supine compared with upright posture (DiMenna et al. 2010), thus, compromising exercising muscle perfusion pressure and O_2_ delivery (Egaña and Green 2007, 2005), which was also accompanied by a significant reduction in V̇O_2peak_ in the supine posture.

Whether older individuals with T2D also demonstrate a higher reliance on fractional oxygen extraction during incremental cycling exercise is currently unknown. This is relevant given that the prevalence of T2D increases substantially with age, and in older individuals with T2D moderate-intensity submaximal exercise seems to be less impacted than in younger/middle-aged individuals (Wilkerson et al. 2011; O'Connor et al. 2015). Specifically, in contrast to the blunted V̇O_2_ kinetics responses observed during submaximal exercise in middle-aged individuals with T2D compared with age-matched controls (Mac Ananey et al. 2011; Bauer et al. 2007; O'Connor et al. 2015, 2012; Kiely et al. 2015), V̇O_2_ kinetics and systemic vascular conductance responses have been shown to be not different in older people with and without T2D (Wilkerson et al. 2011; O'Connor et al. 2015), although in the study by Wilkerson et al. (Wilkerson et al. 2011) a larger change in muscle deoxygenation per V̇O_2_ unit was observed in the older group with T2D. This could be interpreted as them having a greater O_2_ extraction during exercise due to a compromised muscle blood flow. Accordingly, the aim of the present study was to investigate muscle deoxygenation responses during a ramp incremental cycling exercise test to exhaustion in older adults (age range between 60 to 72 yr) with T2D to explore whether blunted microvascular blood flow responses contribute to the reduction in V̇O_2peak_ in these individuals. We hypothesized that older individuals with T2D would have a reduced V̇O_2peak_ and a higher rate of muscle fractional O_2_ extraction compared to age-matched healthy controls.

## Methods

### Participants

Twenty-four white Caucasian individuals, 12 with uncomplicated T2D (8 males, 4 females), and 12 activity level- age- and BMI-matched controls (7 males, 5 females) participated in this study. The age range of all participants was between 60 and 72 yr. Participants in the control group were recruited from the general population, whilst participants with T2D were recruited from the Diabetes Outpatient Clinics of St. Columcille’s and St. Vincent’s University Hospitals (Dublin). All female participants were postmenopausal. All participants were non-smokers and had not smoked during the 12-month period preceding the study. Individuals with T2D had a clinical history of diabetes of between 4 to 16 years (mean: 8.6 ± 4.2 yr), with adequately controlled HbA_1c_ levels (< 10%) and were not taking insulin or beta-blockers. Three of the controls were on prescriptive medications (statins), and with the exception of one participant, all participants with T2D were taking oral hypoglycaemic medication (Table 1). Individuals with T2D displayed no clinical evidence of ischemic heart disease (normal ECG during treadmill stress test following the Bruce protocol), peripheral arterial disease (0.9 < Ankle-Brachial Index, ABI, < 1.3), kidney dysfunction (consistent urinary protein > 200 mg^.^dl^−1^), or liver dysfunction (urinary creatinine levels > 2.2 mg^.^dl^−1^). Participants were classified as physically inactive by self-report [(≤ 150 min.week^−1^ of moderate-intensity (< ventilatory threshold, VT) exercise in the preceding 6 months] which was confirmed by way of RT3 triaxial accelerometers (Stayhealthy Inc, CA) worn over a 5-day period (Rowlands et al. 2004). Levels of inactive/sedentary behaviour (< 1.5 metabolic equivalents or METs), light activity (1.5–3 METs) or moderate-to-vigorous physical activity (> 3 MET) were not different between groups. All participants provided written informed consent before commencement. The study was approved by the Faculty of Health Sciences’ Research Ethics Committee, Trinity College Dublin, and St Vincent’s Healthcare Ethics and Medical Research Committee, and conducted in accordance with the principles outlined by the Declaration of Helsinki.

**Table 1:** Physical characteristics, medication and peak physiological responses to the ramp incremental test

	Control	Type 2 diabetes	*P* value
*n*	12	12	
Physical characteristics			
Sex (male, female), *n*	8, 4	7, 5	
Age, yr	65 ± 4	63 ± 3	0.33
Stature, m	1.69 ± 0.09	1.69 ± 0.06	0.97
BMI, kg/m^2^	26.5 ± 2.6	28.6 ± 3.9	0.15
Body Mass, kg	76.4 ± 11.9	82.3 ± 14.3	0.28
HbA_1c_, %	5.3 (0.3)*	7.5 (2.4)	< 0.01
Fat layer of VL, mm	5.9 ± 2.9	6.8 ± 3.2	0.31
Time since diagnosis, yr		8.6 ± 4.2	
Diabetes medication			
Metformin, *n*		12	
Sulfonylurea, *n*		8	
DPP-4 inhibitor, *n*		4	
Anti-hypertensive medication			
Angiotensin converting enzyme inhibitor, *n*		3	
Angiotensin II receptor blocker, *n*		1	
Statins, *n*	3	6	
Aspirin, *n*		1	
Peak physiological responses			
V̇O_2peak_, L/min	2.18 ± 0.59	1.87 ± 0.35	0.14
V̇O_2peak_, mL.kg^−1^.min^−1^	28.3 ± 5.3*	23.0 ± 4.2	0.01
PO_peak_, W	169 ± 50*	135 ± 31	0.05
Peak HR (beats/min)	150 ± 13	154 ± 16	0.84
MRT, s	60.4 ± 56.4	70.8 ± 52.8	0.63

Data are means ± SD for variables that were normally distributed and median with interquartile range in parentheses for variables that showed significant skewness and were not normally distributed in one or both groups. *n*, no. of participants. *BMI* body mass index, *HbA*_*1c*_ glycosylated haemoglobin, *VL* vastus lateralis, *DPP-4* Dipeptidyl-peptidase 4, *MRT* mean response time, *V̇O*_*2*_ oxygen uptake, *PO* power output, *HR* heart rate

*Significantly different from type 2 diabetes (*P* < 0.05)

### Study protocol

#### Overview

Following satisfactory completion of the 12-lead ECG stress test, participants with T2D were tested on one occasion in the exercise testing facility in St. Columcille’s Hospital, whereas the controls did so in the cardiovascular performance laboratory in the Department of Physiology, Trinity College Dublin. All participants refrained from consuming alcohol, caffeine and non-prescribed nutritional supplements in the 24 h prior to testing and constrained their exercise to normal activities of daily living. All participants performed a ramp incremental cycling test to volitional exhaustion to determine V̇O_2peak_. While participants were tested in different testing facilities for safety precautions, the equipment used was the same in both sites.

#### Ramp incremental cycling test to exhaustion

The ramp incremental cycling test to exhaustion was performed in an upright position on an electrically braked cycle ergometer (Excalibur Sport; Lode B.V., Groningen, The Netherlands). The test started with an initial workload of 10 W for 2 min (i.e., ‘unloaded’ cycling). This was followed by 10–15 W/min increments in PO for females or 10–20 W/min increments in males, depending on stated activity levels, until volitional exhaustion. Pedal frequency was held constant at an individually selected cadence between 60–75 revolutions per minute (rpm). Failure in a test was determined as a drop in cadence exceeding 3 rpm for > 5 s. Peak workload was determined according to the point of termination of the test. V̇O_2peak_ was determined by identifying the highest 15-s mean V̇O_2_ value recorded before the participant’s volitional termination of the test.

### Measurements

#### Pulmonary gas exchange and heart rate

During exercise participants wore a facemask to continuously collect expired air using an online metabolic system (Innocor, Innovision A/S, Odense, Denmark). Analysis of expired air allowed determination of pulmonary O_2_ uptake (V̇O_2_), carbon dioxide output (V̇CO_2_), minute ventilation (V̇_E_) and the respiratory exchange ratio (RER) breath-by-breath. Heart rate was recorded every 5 s (Polar S610i, Polar Ltd, Finland), with peak HR defined as the highest heart rate attained within the last 15 s of the point of termination of the test.

#### Muscle deoxygenation

A continuous wave NIRS system (Hamamatsu Niro 200Nx; Hamamatsu Photonics, Hamamatsu, Japan), was used to non-invasively determine the oxygenation status of the right quadricep’s VL muscle. This was determined using the spatially resolved spectroscopy technique and modified Beer-Lambert principle with three wavelengths of emitting light (λ = 735, 810, and 850 nm). The theoretical basis of NIRS and its use in exercise measurements have been described in detail elsewhere (Ferrari et al. 2011). Briefly, this technique estimates the optical density changes of deoxygenated haemoglobin and myoglobin (HHb + Mb) based on the O_2_ dependency of absorption changes for near-infrared light in these proteins. As the VL muscle is a dominant locomotor muscle during cycling, the present study examined the Δ[HHb + Mb] profiles of the right VL muscle. After shaving the skin, the probes were placed on the belly of the muscle, 10–16 cm above the lateral femoral condyle, parallel to the major axis of the thigh with a 3 cm spacing between the emitter and receiver. The probes were housed in a black rubber holder and secured on the skin surface with bi-adhesive tape and then covered with a dark elastic bandage, which minimised extraneous movement and the intrusion of stray light throughout the exercise protocol. Since the depth of the measured area is estimated to be between one-half and one-third of the distance between the emitter and the receiver, in the present study the thickness of the skin and adipose tissue at the site of the probe placement was measured via 2D ultrasound operating in B-mode (Zonare Ultra Smart Cart, Software version 4.7, USA). This was to ensure that data largely represented absorption of near-infrared light in muscle tissue and not in subcutaneous fat. This was confirmed with all participants having less than 1.5 cm of adipose tissue thickness at the probe location (Table 1).

### Data analysis

#### Muscle deoxygenation

The NIRS-derived signal was normalised whereby the ‘unloaded’ exercise baseline value was adjusted to zero (‘zero set’). Thus, the NIRS data are presented as a relative change from the baseline- to the end-exercise values. As such 0% represents the mean steady-state value of the last 30 s of the unloaded cycling and 100% represents the highest mean value of the last 30 s of any work rate. This was done given the uncertainty of the optical path length in the VL at rest and during exercise, so, data are presented as normalised delta units Δ[HHb + Mb]. The [HHb + Mb] data was collected at a frequency of 1 Hz, was time averaged into 5 s bins for each participant and was normalised to the peak amplitude of the response (Δ%[HHb + Mb]). The [HHb + Mb] response dynamics were expressed in relation to absolute (W) and relative power output (%PO_peak_) prior to curve fitting. Therefore, individual profiles were plotted as a function of Watts and %PO_peak_ and characterised by a linear function with two terms to establish the slope of increase of deoxygenation (slope_1_), plateau as maximal exercise was approached (slope_2_), and the break point (BP) located between the increasing deoxygenation and its plateau. The double linear function was applied using TableCurve 2D (Systat Software, USA) as:

$$y \, = \, a \, + \, b \, \times \, x \, {-} \, c \, \times \left( {x - d} \right) \times f$$

$$f \, = \, if \, \left( {x \, < \, d, \, 0, \, 1} \right)$$

where *a* and *b* represent the y-intercept and slope of the first linear function (slope_1_), *d* is the break point (BP*)* where the segments intersect, with the slope of the second linear function (slope_2_) being calculated from the parameter estimates of *b* and *c* (slope_2_ = *b—c*).

*ΔV̇O*_*2*_*/ΔPO*. The rate of change of V̇O_2_ relative to PO during ramp incremental exercise reflects the capacity of aerobic metabolism to adjust to the non-steady state conditions incurred during a ramp incremental protocol. Initially, the mean response time (MRT) of V̇O_2_ during the ramp incremental exercise was estimated using the approach described by Iannetta et al. (2019) (Iannetta et al. 2019). Briefly, we determined the average steady state V̇O_2_ corresponding to three separate bouts of moderate-intensity constant-power outputs (performed on a separate visit), and we then compared the ramp-derived power output associated with that V̇O_2_ to the constant-power output which elicited that V̇O_2_ (Iannetta et al. 2019). The difference between these power outputs was then converted to the time (taking into account the slope of the ramp protocol) to retrieve the time-interval corresponding to MRT. The breath by breath V̇O_2_ data were averaged over 15 s intervals and plotted as a function of work rate after accounting for the MRT to reflect the increase in aerobic metabolism (ΔV̇O_2_) for each increase in power output (ΔPO). From this the ΔV̇O_2_/ΔPO slope was calculated over the same range of PO as used to determine the first Δ% [HHb + Mb]/PO slope (i.e. parameter *b* or slope_1_) as described above.

#### Statistical analyses

Prior to analysis, normality of the data was assessed using the Shapiro–Wilk’s test. Physical characteristics, activity levels, peak exercise responses and NIRS-derived muscle deoxygenation responses between groups were compared using unpaired 2-tailed Student’s t-test for parametric analyses, or the Mann Whitney U test for non-parametric analyses. Correlations between variables were assessed using the Pearson product-moment correlation coefficient (Pearson r). Statistical significance was set at a *P* ≤ 0.05. All values are expressed as means ± standard deviation (SD) or as median and interquartile ranges for data that were deemed not normally distributed.

## Results

### Physical characteristics

Participants’ physical characteristics and activity levels are shown in Table 1. Both groups were matched according to sex, age, body mass and BMI, but participants with T2D displayed higher HbA_1c_ levels.

### Peak physiological responses from ramp incremental cycling test

Peak physiological responses during the ramp cycling exercise are summarised in Table 1. Values of V̇O_2peak_ normalised to body mass as well as absolute PO_peak_ values were significantly lower in older individuals with T2D compared with controls. Absolute V̇O_2peak_ values were numerically lower in T2D but they were not significantly different.

### NIRS-derived [HHb + Mb] response dynamics and correlations

**Fig. 1:**
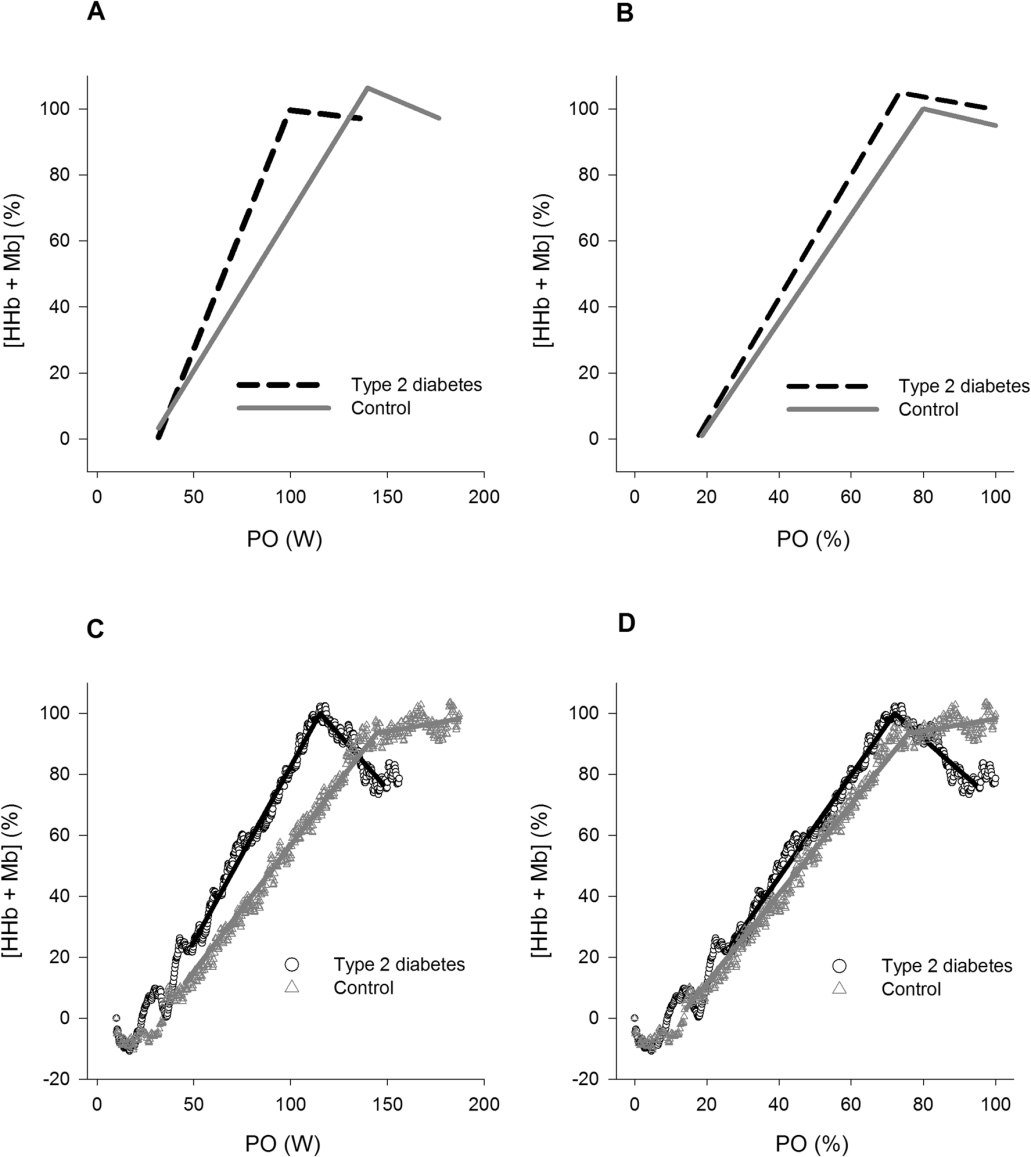
Group mean and individual representative profiles of the modelled %[HHb + Mb] response dynamics during ramp incremental cycling exercise for older individuals with T2D and controls when expressed as a function of absolute power output (mean: **A**, individual: **C**) and relative power output (PO%) (mean: **B**, individual: **D**). For the representative profiles double-linear regression models are superimposed on the data (T2D: black line; Control: grey line). Note the steeper slope_1_ of the %Δ[HHb + Mb]/PO relationship in T2D (**A** and **C**), and not different slope_1_ of the %Δ[HHb + Mb]/PO relationship in both groups (**B** and **D**). The double-linear models in A and B were recreated from the parameters shown in Table 2

**Fig. 2:**
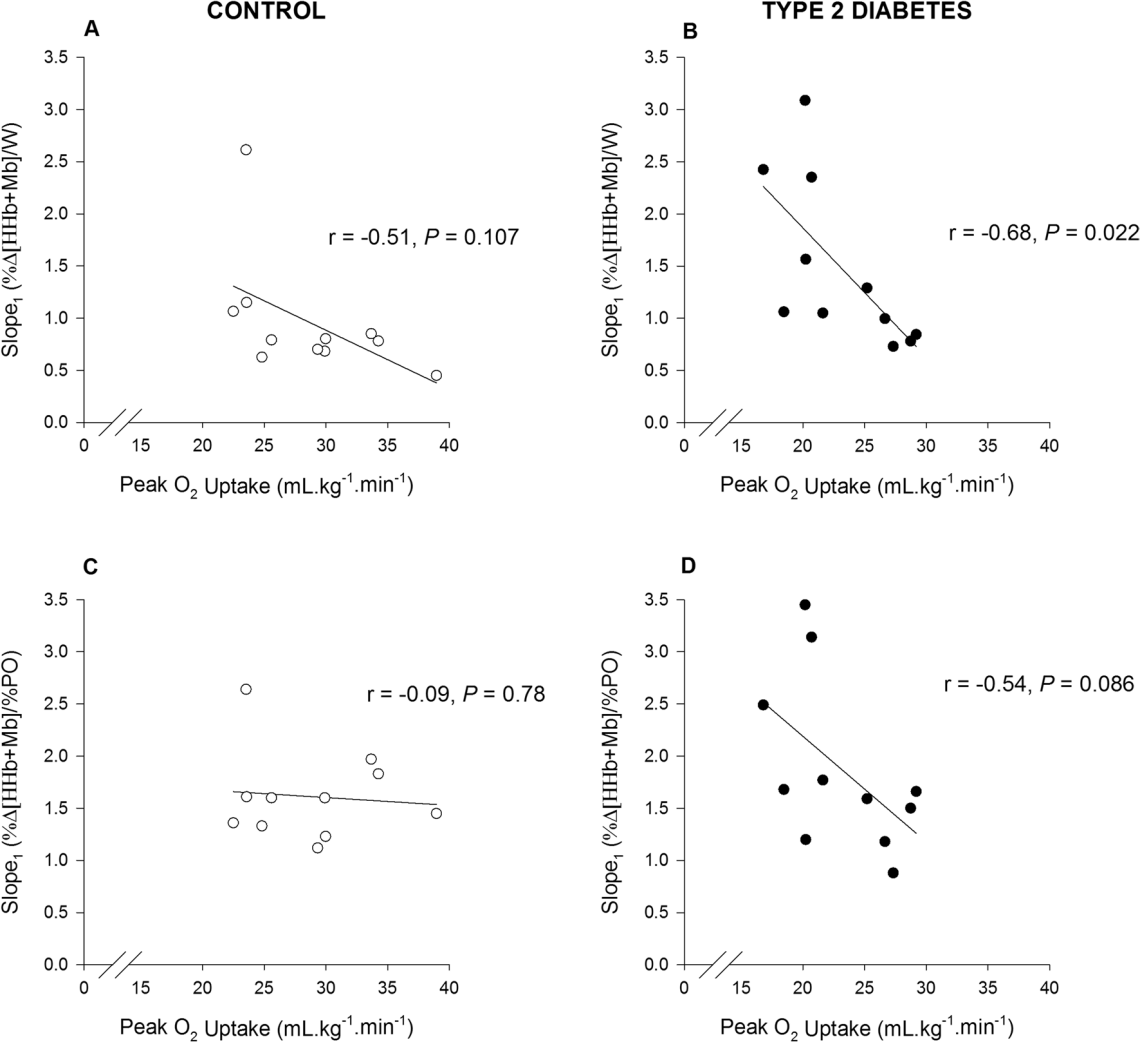
Relationship between the first slope (slope 1) of the %Δ[HHb + Mb]/PO (**A** and **B**) and %Δ[HHb + Mb]/%PO (**C** and **D**) of the double linear regression and V̇O_2peak_ (mL.kg^−1^.min^−1^) in participants with type 2 diabetes and controls

Group mean parameter estimates from the double linear model of the %Δ[HHb + Mb] profile as a function of absolute power output (W) and normalised power output (% PO_peak_) are displayed in Table 2. Mean group profiles and individual representative profiles of the modelled [HHb + Mb] response dynamics as a function of PO and %PO are displayed in Fig. 1, while the correlations between slope_1_ and relative V̇O_2peak_ for both groups are shown in Fig. 2. Due to a technical error with the NIRS data, responses from one male with T2D and one control female were excluded from the analysis. The slope_1,_ used to establish the dynamic adjustment of [HHb + Mb] as a function of PO (W) was greater in T2D than controls, while the BP was lower in T2D than controls, and slope_2_ was not different between groups (Table 2, Fig. 1). Slope_1_ was significantly correlated with V̇O_2peak_ (ml.kg^−1^.min^−1^) in T2D, but not controls (Fig. 2). When the relationship of the Δ%[HHb + Mb] profile was expressed as a function of %PO no differences were observed in slope_1,_ slope_2_ or the BP between groups; and slope_1_ was not correlated with V̇O_2peak_ (ml.kg^−1^.min^−1^) in T2D nor controls (Fig. 2).

**Table 2:** Parameter estimates for the %Δ[HHb + Mb] profile for both groups plotted as a function of absolute PO (W) and normalised PO (% PO_peak_) during the ramp incremental test

	Control	Type 2 diabetes	*P *value
PO (W)			
Slope_1_	0.79 (1.06)	1.06 (1.50)*	0.04
Slope_2_	− 0.26 ± 0.29	− 0.06 ± 0.59	0.32
BP (W)	140 ± 34	101 ± 35*	0.01
PO (%)			
Slope_1_	1.60 (0.65)	1.66 (1.29)	0.65
Slope_2_	0.01 (0.48)	0.09 (0.31)	0.15
BP (%)	79 ± 13	74 ± 19	0.44

*Significantly different from control (*P* < 0.05)

Data are means ± SD for variables that were normally distributed and median with interquartile range in parentheses for variables that showed significant skewness and were not normally distributed in one or both groups. Slope_1_ and slope_2_ of linear regression before and after break point (BP), respectively

### ***ΔV̇O***_***2***_***/ΔPO***

The rate of change in V̇O_2_/PO was not significantly different during the ramp incremental exercise test between the older groups with T2D and controls, with no observed differences in the slopes (10.5 ± 1.5 vs. 9.5 ± 1.2 mL.min^−1^.W^−1^ respectively, *P* = 0.36).

## Discussion

The principal finding of the current study was that the primary slope of the bi-linear regression model used to establish the dynamic adjustment of muscle deoxygenation for a given absolute power output (or V̇O_2_) during a ramp incremental cycling test was larger in sedentary older individuals with uncomplicated T2D compared with activity-, age- and BMI-matched controls, which was accompanied with significant reduction in V̇O_2peak_ and PO_peak_ in participants with T2D. Thus, our findings suggest a dysfunction within the O_2_ delivery to utilization balance within the skeletal muscle of older individuals with T2D, in keeping with previously reported larger Δ[HHb]/Δ V̇O_2_ during submaximal exercise transitions (Wilkerson et al. 2011), and as such, likely influenced the reduced V̇O_2peak_ responses observed. Herein, all participants were inactive/sedentary, and physical activity levels did not differ between groups. This suggests that the reduced V̇O_2peak_ response, which is in line with previous observations in older and younger/middle-aged individuals with uncomplicated T2D (see introduction), was likely not affected by differences in activity levels.

In the present study the profile of Δ%[HHb + Mb] was characterised by a bi-linear regression model which provides an enhanced understanding of the dynamic balance between regional oxygen delivery and V̇O_2_ at the level of the microvasculature (Spencer et al. 2012). In the first linear component an increase in Δ%[HHb + Mb] relative to changes in work rate occurs, denoting the increasing reliance on O_2_ extraction relative to metabolic demand. This segment terminates at a “break point” (Δ%[HHb + Mb] – BP), from which a “plateau-like” second linear component evolves even if the work rate continues to increase. Importantly, this break point does not indicate the upper limit of O_2_ extraction, and it seems to be associated with the transition between the heavy- and severe-exercise intensity domains (Keir et al. 2015). Once this upper boundary of exercise is achieved, the [HHb + Mb] plateau signal has been shown to be related to the redistribution of blood flow to active muscles (Inglis et al. 2017). In the present study, the primary slope of the Δ%[HHb + Mb]/ΔPO response (slope_1_) during the ramp test was greater in older individuals with T2D inferring that O_2_ availability within the microvasculature of the exercising muscle is likely limited in T2D, which may indicate a poor matching of O_2_ delivery and utilisation within the active musculature to meet the muscles V̇O_2_ demands for exercise. Supporting this notion, profound impairments and altered O_2_ delivery-utilization dynamics have been observed in rodent models of T2D impacting microvascular oxygenation profiles (Padilla et al. 2007, 2006). Specifically, significant reductions in the percentage of flowing capillaries and microvascular partial pressure of O_2_ (P*mv*o_2_) at rest (Padilla et al. 2006) and during electrically stimulated muscle contractions (Padilla et al. 2007) were reported compared with healthy controls, eliciting a reduction in O_2_ diffusion capacity across the capillary-myocyte space to the mitochondria (Behnke et al. 2002; Padilla et al. 2007, 2006), These altered profiles of muscle fractional extraction in T2D appear to be mediated, at least partly, by impairments in endothelium-dependent vasodilation of resistance vessels (Kingwell et al. 2003).

Reductions in O_2_ availability within the microvasculature of the exercising muscle are also evidenced when a ramp exercise is carried out in the supine posture (DiMenna et al. 2010). Therein, DiMenna and colleagues observed a leftward shift in the Δ%[HHb + Mb]/ Δ%PO slope of a sigmoidal curve when active young participants cycled in the supine compared to upright posture, which is indicative of an over reliance on O_2_ extraction for a given power output attributed to a loss of gravity-enhanced perfusion pressure in the active muscles (Egaña et al. 2010b, 2010a, 2013; Jones et al. 2006). In contrast, trained healthy individuals demonstrate a rightward shift in the Δ%[HHb + Mb]/Δ%PO slope of a sigmoidal curve compared with untrained counterparts likely due to an enhanced oxidative capacity and/or larger proportion of slow-twitch fibres (Boone et al. 2009). In fact, our laboratory has recently shown in participants with T2D that improvements in V̇O_2peak_ during a ramp cycling exercise following both, continuous and intermittent aerobic exercise training were accompanied by significant reductions in the slope_1_ of the bilinear regression of the Δ%[HHb + Mb]/Δ%PO response in T2D (Gildea et al. 2021a), suggesting an exercise training-induced improved microvascular O_2_ delivery, which was also evident during submaximal exercise transitions (Gildea et al. 2022, 2021b). On the other hand, Gravelle and colleagues (Gravelle et al. 2012) reported that older recreationally active males had a greater slope in the Δ%[HHb + Mb] response relative to absolute power output of a sigmoidal curve (i.e. a leftward shift in the Δ%[HHb + Mb]/ΔPO slope) during a ramp cycling exercise compared with young active males accompanied with a reduced V̇O_2peak_ and PO_peak_ (Gravelle et al. 2012). Therein, when Δ%[HHHb + Mb] was expressed as relative power output (Δ%PO) to account for their lower PO_peak_ in the ramp test no difference in the slope of the sigmoid fit was observed between groups. Authors suggested that at an equivalent relative exercise intensity the interplay between sympathetic activation, parasympathetic withdrawal, and the generation of vasodilatory metabolites from the active muscles lead to a comparable dependence on muscle oxygen extraction in both groups.

In the present study, when the Δ%[HHb + Mb] was expressed as a function of %PO_peak_, the primary slope (slope_1_) of the bi-linear function was not different between the two sedentary older groups (T2D (median (interquartile range)): 1.66 (1.29); controls: (1.60 (0.60)). However, this slope_1_ was larger in middle-aged sedentary individuals with T2D (1.59 (1.14) with similar diabetes characteristics recruited from the same community than in sedentary middle-aged controls (1.23 (0.51) (Gildea et al. 2019). In addition, even if herein the slope_1_ of the Δ%[HHb + Mb] response and absolute PO was inversely correlated with V̇O_2peak_ among older participants with T2D, when this relationship was expressed as a function of %PO_peak_ the correlation did not reach significance (*P* = 0.087), whereas slope_1_ of the Δ%[HHb + Mb]/Δ%PO response was significantly correlated with V̇O_2peak_ among middle-aged individuals with T2D (Gildea et al. 2019). Collectively, these data imply that microvascular O_2_ delivery is likely impaired during ramp incremental exercise in both, middle-aged and older sedentary individuals with T2D, but that the magnitude of this effect is greater among middle-aged participants with T2D compared with age-matched controls than in older individuals with T2D versus their counterpart controls. While further studies are needed to better understand how these diabetes-related impairments differ among individuals of different age groups, the reductions in systemic vascular conductance responses observed in middle-aged individuals with T2D compared with controls that were absent among the older individuals with and without T2D (O'Connor et al. 2015) likely contribute, at least partly, to these effects.

In addition to the findings in the present study of greater muscle fractional O_2_ extraction in older participants with T2D compared with controls up to the Δ[HHb + Mb]BP or ~ 75% PO_peak_, it is possible that limitations in microvascular blood flow at intensities above the Δ[HHb + Mb] break point are also present in older individuals with T2D compared with older controls. For instance, peak leg blood flow and vasodilatory capacity during maximal graded calf plantar-flexion exercise were shown to be attenuated in adults with T2D (mean age 57 yr) than controls, which were accompanied by a lower peak force relative to maximal voluntary contraction during the calf graded test, as well as reduced V̇O_2peak_ and PO_peak_ during a graded cycling test (Kiely et al. 2014). In contrast, in healthy inactive/sedentary younger (mean age 23 yr) and older (mean age 67 yr) adults peak leg blood flow and vascular conductance responses were not different, while the peak force at the end of the calf graded test was reduced in the older groups (Reilly et al. 2018). Thus, reductions in O_2_ delivery near maximal exercise intensities in older individuals with T2D compared with older but otherwise healthy counterparts may also account for the reductions in exercise tolerance observed in the older group with T2D. Other additional factors possibly influencing the reduced V̇O_2peak_ in older individuals with T2D include impairments in cardiac function (Wilson et al. 2017a, 2017b), alterations in muscle fibre type distribution towards a more glycolytic phenotype (Marin et al. 1994) as well as reductions in mitochondrial content and/or functional capacity (Ritov et al. 2005; Boushel et al. 2007).

## Limitations

The present study involved the performance of a single incremental exercise test. While the observed lower V̇O_2peak_ responses in individuals with T2D compared to healthy controls align with previously documented declines in V̇O_2peak_ or V̇O_2max_ in adults with T2D (Green et al. 2015), to enhance the confidence in the outcome, future studies should conduct an additional incremental test and/or verification test. It is also pertinent to acknowledge that the deoxygenated data presented here were limited to one superficial muscle, and that the NIRS technology used herein reveals temporal and spatial heterogeneity among active muscles in the leg as well as within the vastus lateralis (Okushima et al. 2015; Heinonen et al. 2015; Koga et al. 2007). Our results are limited to mixed groups of men and women with a slightly different male/female ratio; hence, further studies should explore sex-related differences in these outcomes. Given that the majority of participants were male, we reanalysed the data excluding the female participant and the main statistical outcomes were unaffected. It is therefore likely that the current findings are applicable among male participants.

## Conclusions

In conclusion, older participants with T2D showed a reduced V̇O_2peak_ compared with healthy controls during an incremental ramp cycling exercise, accompanied by a steeper slope of the first linear component of the bi-linear Δ%[HHb + Mb]/ΔPO response. These findings suggest that a greater rate of fractional O_2_ extraction in the exercising musculature for a given increase in absolute PO up to the point of the muscle deoxygenation break point, indicative of reduced adjustments in microvascular O_2_ delivery, is likely a contributing factor in the reduction of V̇O_2peak_ observed in older individuals with T2D compared to age-matched controls.

## Data Availability

Data are available upon reasonable request from the corresponding author.

## Abbreviations:

a-vO_2diff_: Arteriovenous oxygen difference
BP: Break point
HR: Heart rate
HHb + Mb: Deoxygenated haemoglobin and myoglobin
MRT: Mean response time
NIRS: Near-infrared spectroscopy
PO: Power output
T2D: Type 2 diabetes
V̇O_2_: Oxygen uptake
V̇O_2peak_: Peak oxygen uptake
VT: Ventilatory threshold
VL: Vastus lateralis
